# Safety Evaluation of *Neo* Transgenic Pigs by Studying Changes in Gut Microbiota Using High-Throughput Sequencing Technology

**DOI:** 10.1371/journal.pone.0150937

**Published:** 2016-03-11

**Authors:** Qingqing Wang, Lili Qian, Shengwang Jiang, Chunbo Cai, Dezun Ma, Pengfei Gao, Hegang Li, Ke Jiang, Maoxue Tang, Jian Hou, Jie Liu, Wentao Cui

**Affiliations:** 1 Institute of Animal Sciences, Chinese Academy of Agricultural Sciences, Beijing, 100193, P R China; 2 State Key Laboratory of Agrobiotechnology, China Agricultural University, Beijing, 100193, P R China; 3 Institute of Animal Sciences, Qingdao, 266100, P R China; 4 Department of Bioengineering and Biotechnology, College of Chemical Engineering, Qingdao University of Science & Technology, Qingdao, 266042, P R China; Wageningen University, NETHERLANDS

## Abstract

The *neo* (neomycin phosphotransferase) gene is widely used as a selection marker in the production of genetically engineered animals and plants. Recent attention has been focused on safety concerns regarding *neo* transgene expression. In this study, *neo* transgenic and non-transgenic piglets were randomly assigned into Group A and Group B to evaluate effects of *neo* transgene by studying changes in gut microbiota using high-throughput sequencing. Group A pigs were fed a standard diet supplemented with antibiotic neomycin; Group B pigs were fed a standard diet. We examined horizontal transfer of exogenous *neo* gene using multiplex PCR; and investigated if the presence of secreted NPT II (*neo* expression product) in the intestine could lead to some protection against neomycin in transgenic pigs by monitoring different patterns of changes in gut microbiota in Group A animals. The unintended effects of *neo* transgene on gut microbiota were studied in Group B animals. Horizontal gene transfer was not detected in gut microbiota of any transgenic pigs. In Group A, a significant difference was observed between transgenic pigs and non-transgenic pigs in pattern of changes in *Proteobacteria* populations in fecal samples during and post neomycin feeding. In Group B, there were significant differences in the relative abundance of phyla *Firmicutes*, *Bacteroidetes* and *Proteobacteria*, and genera *Lactobacillus* and *Escherichia-Shigella-Hafnia* between transgenic pigs and non-transgenic pigs. We speculate that the secretion of NPT II from transgenic tissues/cells into gut microbiota results in the inhibition of neomycin activity and the different patterns of changes in bacterial populations. Furthermore, the *neo* gene also leads to unintended effects on gut microbiota in transgenic pigs that were fed with basic diet (not supplemented with neomycin). Thus, our data in this study caution that wide use of the *neo* transgene in genetically engineered animals should be carefully considered and fully assessed.

## Introduction

The neomycin phosphotransferase gene (*neo*) is located on transposon Tn5[1]. It encodes the aminoglycoside 3'-phosphotransferase (denoted *aph*(3')-II or NPT II) enzyme, which inactivates a range of aminoglycoside antibiotics such as kanamycin, neomycin, geneticin (G418), and paromomycin by phosphorylation[2]. *neo* is the most widely used selectable marker in preparing transgenic animals and plants [3–5]. Therefore, attention has been paid to a variety of side effects and potential safety concerns related to *neo* transgene expression. Particularly, it is necessary to evaluate the effect of *neo* transgene expression on transgenic animals and their environment. Currently, to our best knowledge, almost no reports have been published on safety evaluations of *neo* transgene expression in transgenic animals[6]. Specifically, no study has been conducted to evaluate the direct effects of *neo* transgene expression and the unintended effects on the gut microbiota in transgenic livestock animals.

The dissemination of antibiotic resistance genes by horizontal gene transfer has led to the rapid emergence of antibiotic resistance among bacteria. Integron is an antibiotic resistance gene capture and expression system[7]. Recent studies have shown that integrins are the major cause of antibiotic resistance, particularly in the development of multiple resistance in gram-negative bacteria[8]. In our current study, since transgenic pigs contain exogenous neo gene, theoretically speaking, it is possible that DNA fragments from intestinal shedding cells could be captured and integrated by certain gut microorganisms. Therefore, it is necessary to examine if gene transfer occurs in *neo* transgenic pigs. In the swine industry, aminoglycoside antibiotics such as kanamycin, neomycin and gentamicin are widely used for disease treatment and as food supplements to enhance growth[9]. It is likely that the *neo* transgene expression product NPT II in transgenic pigs can be released from dead epithelial cells into the intestines, where NPT II can inactivate/inhibit a range of aminoglycoside antibiotics such as kanamycin, neomycin, geneticin (G418), and paromomycin by phosphorylation [2]. Thus, it is necessary to evaluate the effect of *neo* transgene expression on gut microbiota and eventually the therapeutic effects of neomycin in transgenic animals.

Unintended effects refer to unexpected effects that cannot be avoided by the integration of a new gene into an organism[10]. The assessment of unintended effects is part of the safety evaluation for transgenic animals. Unintended effects also represent a top international topic that is being investigated using advanced analytical methods or approaches[11]. Potential changes in physiological and metabolic activities of host cells or tissues can be identified or detected without bias using a variety of advanced analytical methods. A stable and balanced gut microbiota plays an important role in the physiological and metabolic activities for human or animal health, and therefore the intestinal microbiota represents a key area in studying unintended effects. The small intestine is not only the first barrier to the environment, bacteria, and food antigens but also the biggest immunological organ[12]. The intestine plays pivotal functional roles in host nutritional metabolism and immunity [13, 14]. There are few studies focusing on the intestinal microbiota to evaluate safety issues related to transgenic animals. Our lab has previously evaluated the effect of the *sFat-1* transgene on intestinal microbiota in transgenic pigs using traditional culture method[15].

It is reported that 60–80% of microbiota cannot be cultured or are very difficult to culture using traditional culture method[16, 17]; thus, the traditional culture method cannot reflect the real relationship between the structure and the bacterial populations inside the intestinal tract. However, pyrophosphate sequencing is a high-throughput method that is rapid, highly accurate and sensitive, automated, and can be reliably used to determine the actual relative abundance of gut microbiota[18–21].

In this study, *neo* transgenic pigs were employed in combination with Roche 454 high-throughput technology to evaluate, for the first time, the effect of *neo* transgene expression on the therapeutic effects of neomycin in transgenic animal and the unintended effects on the gut microbiota. The horizontal gene transfer between animals and gut microbiota was also investigated. We believe that this study will help us to explore the safety issues related to *neo* transgene expression in the transgenic field and to provide scientific data to support regulation by government agencies and understanding by the public of transgenic studies.

## Results

### Identification of *neo* transgenic pigs and expression of *neo* gene

**Fig 1:**
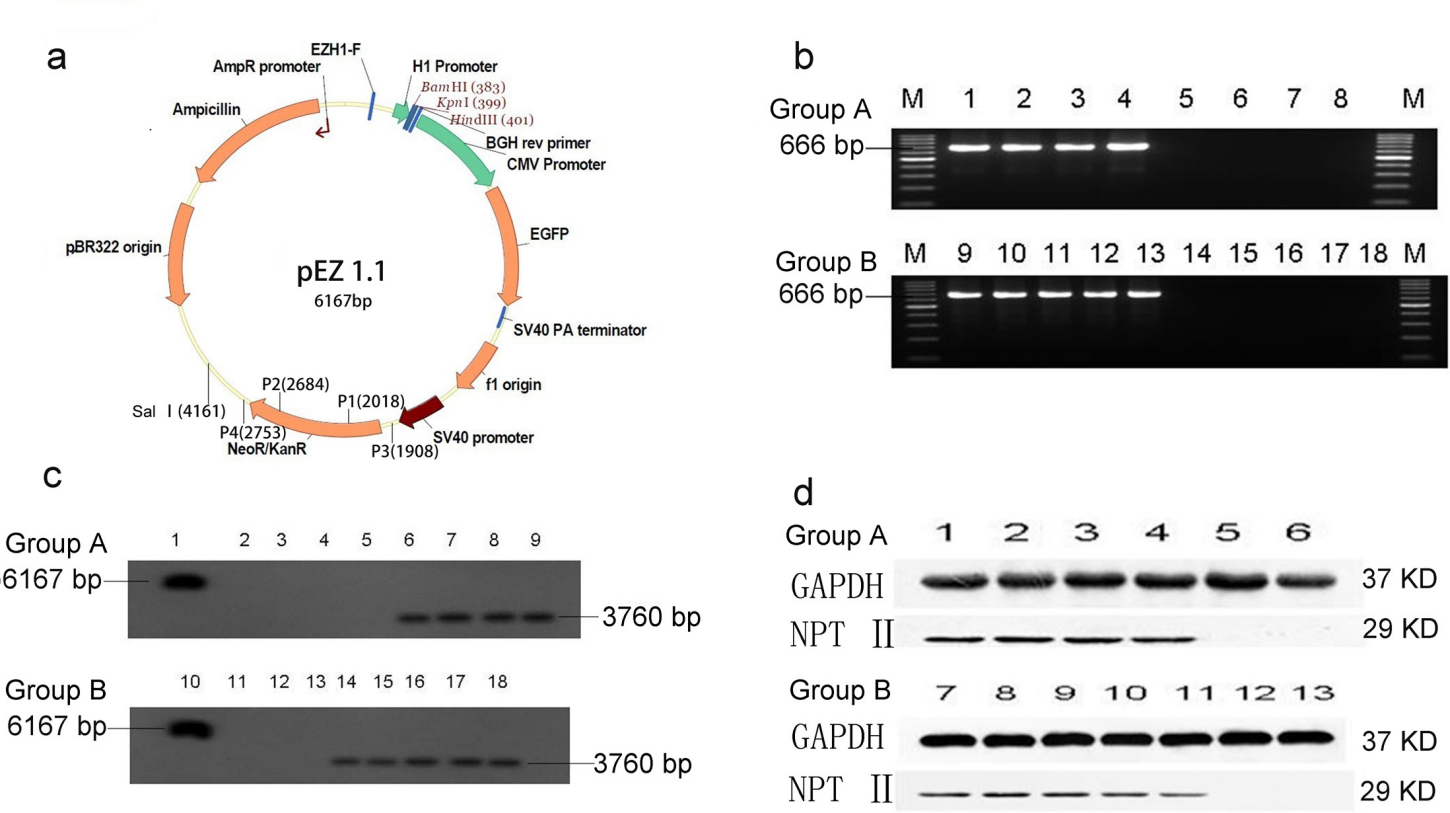
*Neo* transgene integration and expression. a: *Neo* plasmid PEZ1.1. b: PCR detection of *neo* transgene integration in Group A and Group B pigs. The PCR product at 666 bp indicates the integration of *neo* in transgenic pigs (lanes 1–4 and 9–13). No PCR product was detected in non-transgenic pigs (lanes 5–8 and 14–18). c. Southern blot of transgenic (lanes 6–9 and 14–18) and non-transgenic (lanes 2–5 and 11–13) pig samples from Groups A and B. 3760 bp is the length of DNA containing the *neo* gene. d. Western blot of intestinal tissue extracts from transgenic pigs (lanes 1–4 and 7–11) and non-transgenic (lanes 5–6 and 12–13) pigs. Note that the *neo* expression product NPT II was detected as a protein band at 29 kDa in transgenic samples only. The GAPDH band at 37 kDa is the internal reference.

Group A and Group B pigs were generated by mating one *neo* transgenic male pig with two wild type female pigs. Data from PCR and Southern blotting confirmed that there were 4 transgenic and 4 non-transgenic piglets in Group A; 5 transgenic and 5 non-transgenic piglets in Group B (**Fig 1**). Results of Western blotting indicated that tissues from various sections of the intestinal tract (duodenum, jejunum, ileum, cecum, colon and rectum) in transgenic piglets in both Groups A and B expressed high levels of NPT II protein, which is the expression product of *neo* gene. On the other hand, no NPT II was detected in all samples collected from non-transgenic piglets (**Fig 1**). Additionally, we measured NPT II protein concentration in fecal samples using ELISA. Results of ELISA measurement clearly indicated that no NPT II protein was detected in fecal samples from non-transgenic pigs, while nanogram levels (6.2–7.3 ng/ml) of NPT II protein were detected in fecal samples from all transgenic pigs (**Table 1**).

**Table 1:** NPT II concentration in intestinal fecal samples collected from transgenic and non-transgenic pigs measured by ELISA.

Animal ID in Group A	NPT II concentration (ng/ml)	Animal ID in Group B	NPT II concentration (ng/ml)
Negative control	0.0	Negative control	0.0
T1	6.8±0.14	T5	6.2±0.098
T2	7.0±0.12	T6	6.6±0.11
T3	8.3±0.16	T7	5.9±0.12
T4	7.1±0.12	T8	7.2±0.08
NT1	BDL	T9	5.0±0.07
NT2	BDL	NT5	BDL
NT3	BDL	NT6	BDL
NT4	BDL	NT7	BDL
		NT8	BDL
		NT9	BDL

Negative control: PBS; T1-T4: transgenic pigs in Group A; NT1- NT4: non-transgenic pigs in Group A; T5-T9: transgenic pigs in Group B; NT5-NT9: non-transgenic pigs in Group B. BDL: below the detection level.

### Horizontal transfer of *neo* transgene from animal tissues to gut microbiota

**Fig 2:**
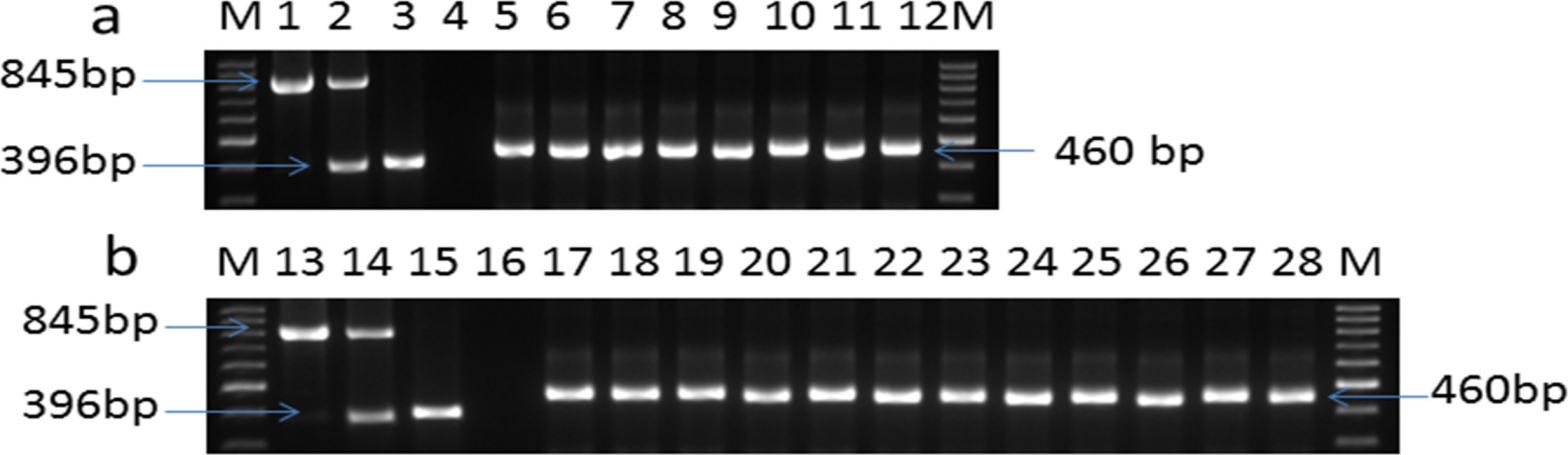
PCR detection of horizontal transfer of the *neo* transgene to the gut microbiota of transgenic pigs. a: Samples from Group A pigs: lane 1, *neo* positive control plasmid; lane 2, ear sample from a transgenic pig; lane 3, ear sample from a non-transgenic pig; lane 4, blank; lanes 5–8, fecal samples from transgenic pigs collected at day 0, day 8, day 23 and day 45 post antibiotic feeding; lanes 9–12, fecal samples from non-transgenic pigs collected at day 0, day 8, day 23 and day 45 post antibiotic feeding. b: Samples from group B pigs: lane 13, *neo* positive control plasmid; lane 14, ear sample from a transgenic pig; lane 15, ear sample from a non-transgenic pig; lane 16, blank; lanes 17–22, fecal samples from the duodenum, jejunum, ileum, cecum, colon, and rectum of transgenic pigs; lanes 23–28, fecal samples from the duodenum, jejunum, ileum, cecum, colon, and rectum of non-transgenic pigs. M: molecular weight markers. 845 bp: PCR product of *neo* gene and SV40 promoter (primers P3 and P4); 396 bp: PCR product of the porcine beta-actin gene (primers P5 and P6); 460 bp: PCR product of 16S rDNA (primers P7 and P8).

In this study, the multiplex PCR method was employed to detect whether horizontal transfer of the *neo* transgene occurred from animal tissues or cells to gut microbiota in the fecal samples collected at different time points post neomycin feeding in Group A and in the fecal samples collected from various sections of the intestinal tract in Group B. According to the multiplex PCR results, with our method, we did not detect the internal reference gene porcine beta-actin (396bp) in any fecal sample extracts, but a band corresponding to 16S rDNA (460bp) was clearly detected, which confirmed that fecal DNA samples collected from the intestinal tract of all pigs were mainly bacterial genome. With our method, no exogenous *neo* gene (the 845bp band) was detectable in samples of microbial genomes (**Fig 2A and 2B**). This result indicated that horizontal transfer of the *neo* transgene from porcine tissues or cells to the microbiota genome is unlikely to occur when PCR was used as a detection method.

### Different patterns and/or magnitudes of changes in phylum and genus levels between transgenic and non-transgenic pigs post neomycin feedings in Group A

**Fig 3:**
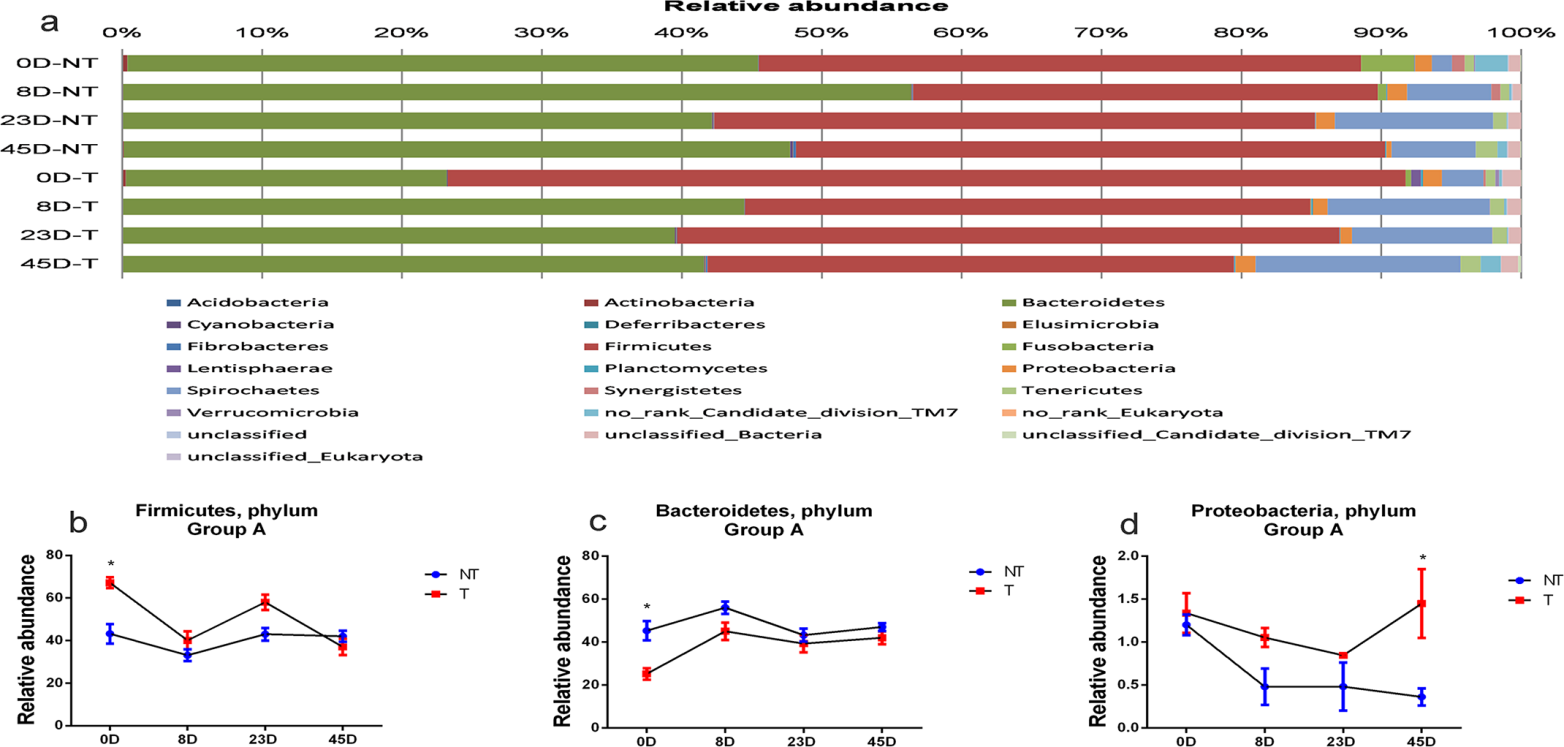
Different patterns in changes in phylum level between transgenic and non-transgenic pigs post neomycin feedings in Group A. Comparison of the relative abundance of bacterial phyla (a) in fecal samples collected from the rectums of transgenic and non-transgenic pigs at various time points (0D, 8D, 23D and 45D) post neomycin feeding; comparison of changes in the levels of *Firmicutes* (b), *Bacteroidetes* (c), and *Proteobacteria* (d) at various time points (0D, 8D, 23 and 45D) post neomycin feeding between transgenic (T) and non-transgenic (NT) pigs in Group A. *: P<0.05.

Statistical analysis revealed 22 phyla in Group A pigs, with *Firmicutes* and *Bacteroidetes* accounting for approximately 95% and all other phyla at very low levels; These findings are consistent with those of previous reports [22, 23]. Comparative analysis indicated that there are differences in the magnitude of the phyla *Firmicutes* and *Bacteroidetes* between transgenic and non-transgenic pigs (**Fig 3A**). For *Firmicutes*, the relative abundance decreased from day 0 to day 8 post neomycin feeding in both transgenic and non-transgenic pigs, but the change was more dramatic in transgenic pigs, decreasing from 67.2% to 40.1% (P-value = 0.042). From day 8 to day 23 post neomycin feeding, the relative abundance of *Firmicutes* increased and was stable in both transgenic and non-transgenic pigs, but the change in transgenic pigs was more dramatic, increasing from 40.1% to 58.0% (P-value = 0.048). From day 23 to day 45 post antibiotic feeding, the relative abundance of the phylum *Firmicutes* decreased from 58.0% to 37.0% (P-value = 0.041) in transgenic pigs, while there were no significant changes (P-value = 0.21) in non-transgenic pigs (**Fig 3B and S1A Fig**). For *Bacteroidetes*, the relative abundance increased in both transgenic and non-transgenic pigs from day 0 to day 8 post neomycin feeding. However, the change in transgenic pigs was more substantial, increasing from 25.2% to 45.1% (P-value = 0.032). From day 8 to day 45 post neomycin feeding, the relative abundance decreased and stabilized in all pigs (**Fig 3C and S1B Fig**).

The phylum *Proteobacteria* comprises Gram-negative bacteria, is found in porcine intestines and is very sensitive to inhibition by neomycin sulfate[24]. Comparative analysis of *Proteobacteria* at different time points post neomycin feeding indicated that there are very different patterns in the changes in levels of *Proteobacteria* between transgenic and non-transgenic pigs. From day 0 to day 8 post antibiotic (neomycin) feeding, although the level of *Proteobacteria* decreased in both non-transgenic and transgenic pigs, the difference in the magnitude was substantial between non-transgenic and transgenic pigs. For example, the relative abundance decreased dramatically, lowering from 1.20% at day 0 to 0.48% (P-value = 0.008) at day 8 in non-transgenic pigs, whereas the decrease in the relative abundance in transgenic pigs was relatively minimal (from 1.34% to 1.05%) (P-value = 0.16). As is clearly shown in Fig 3D, from day 8 to day 23 and from day 23 to day 45 post antibiotic (neomycin) feeding, the relative abundance of *Proteobacteria* in fecal samples from non-transgenic pigs remained lower, while the relative abundance in fecal samples from transgenic pigs decreased from day 8 to day 23 (from 1.05% to 0.84%, P-value = 0.12) and then increased to a normal level (as observed at day 0) at day 45 (**Fig 3D and S1C Fig**).

**Fig 4:**
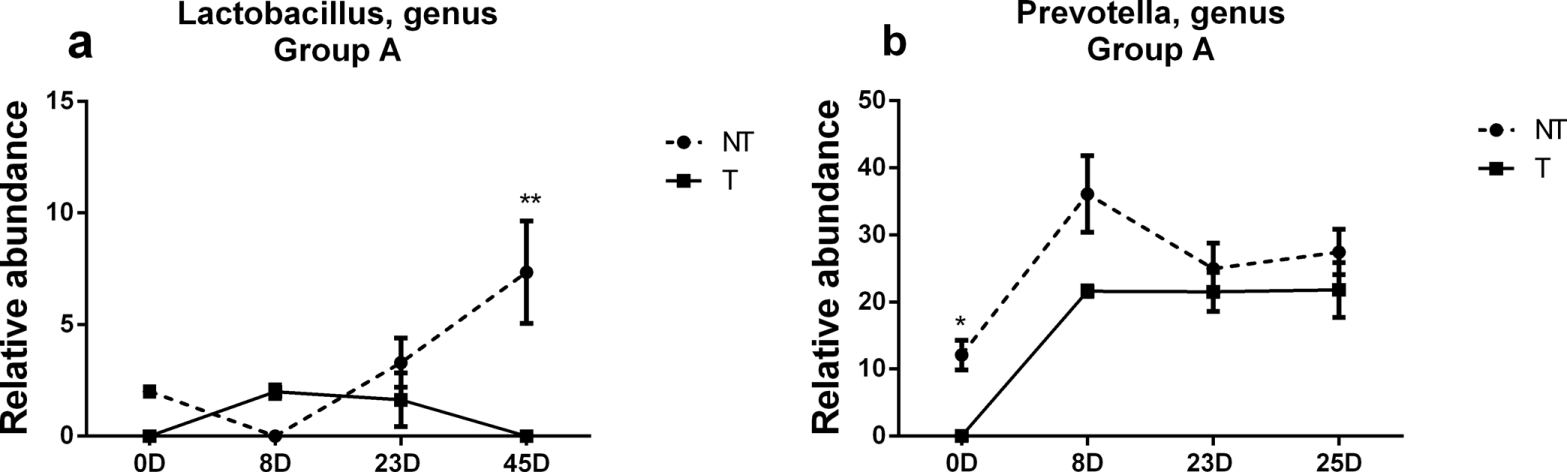
Different patterns in changes in genus level between transgenic and non-transgenic pigs post neomycin feedings in Group A. Comparison of changes in the levels of *Lactobacillus* (a) and genus *Prevotella* (b) at various time points (0D, 8D, 23 and 45D) post neomycin feeding between transgenic (T) and non-transgenic (NT) pigs. *: P<0.05.

In addition, analysis was performed at the genus level for all fecal samples collected from all Group A pigs at different time points post neomycin feeding (**S2 Fig**). Further comparative analysis for the 16 most dominant genera (accounting for 72–89% of the total microbiota) indicated that there were significant differences in magnitude for two genera post neomycin feeding (**S1 Table**). Although the relative abundance of the genus *Lactobacillus* from day 0 to day 8 post neomycin feeding changed dramatically in both transgenic and non-transgenic pigs, the pattern of changes followed an opposite trend, dramatically decreasing from 2% to 0.01% (P-value = 0.008) in non-transgenic pigs but increasing from 0.01% to 1.99% (P-value = 0.008) in transgenic pigs. From day 8 to day 23 post neomycin feeding, the relative abundance of the genus *Lactobacillus* dramatically changed in non-transgenic pigs, increasing from 0.01% to 3.3% (P-value = 0.007), whereas no significant change occurred in transgenic pigs. From day 23 to day 45 post neomycin feeding, the relative abundance of the genus *Lactobacillus* dramatically changed in both transgenic and non-transgenic pigs, but with the opposite trend, increasing from 3.3% to 7.35% (P-value = 0.04) in non-transgenic pigs and decreasing from 1.63% to 0.01% (P-value = 0.009) in transgenic pigs (**Fig 4A and S3A Fig**). The genus *Prevotella* increased in both transgenic and non-transgenic pigs from day 0 to day 8 post neomycin feeding, with the relative abundance increasing from 12.1% to 36.1% (P-value = 0.018) in non-transgenic pigs and increasing from 0.8% to 21.6% (P-value = 0.003) in transgenic pigs. From day 8 to day 23 post neomycin feeding, the relative abundance of *Prevotella* dramatically decreased in non-transgenic pigs (from 36.1% to 25.0%, P-value = 0.05), while there was no obvious change in transgenic pigs. From day 23 to day 45 post neomycin feeding, there was no difference in the changes between transgenic and non-transgenic pigs (**Fig 4B and S3B Fig**).

The overall distribution of bacterial populations was analyzed using Principal Coordinate Analysis (PCoA). Results indicated that bacterial communities in non-transgenic pigs clustered closely to one another at 0D and 45D, while those in transgenic pigs did not. On the other hand, the opposite occurred between transgenic and non-transgenic pigs at 7D and 23D (**S4 Fig**). This observation, to a certain degree, demonstrates that there is a difference in overall distribution of bacterial populations between transgenic and non-transgenic pigs.

### Comparative analysis of phylum and genus levels in various intestinal sections collected from transgenic and non-transgenic pigs in Group B

**Fig 5:**
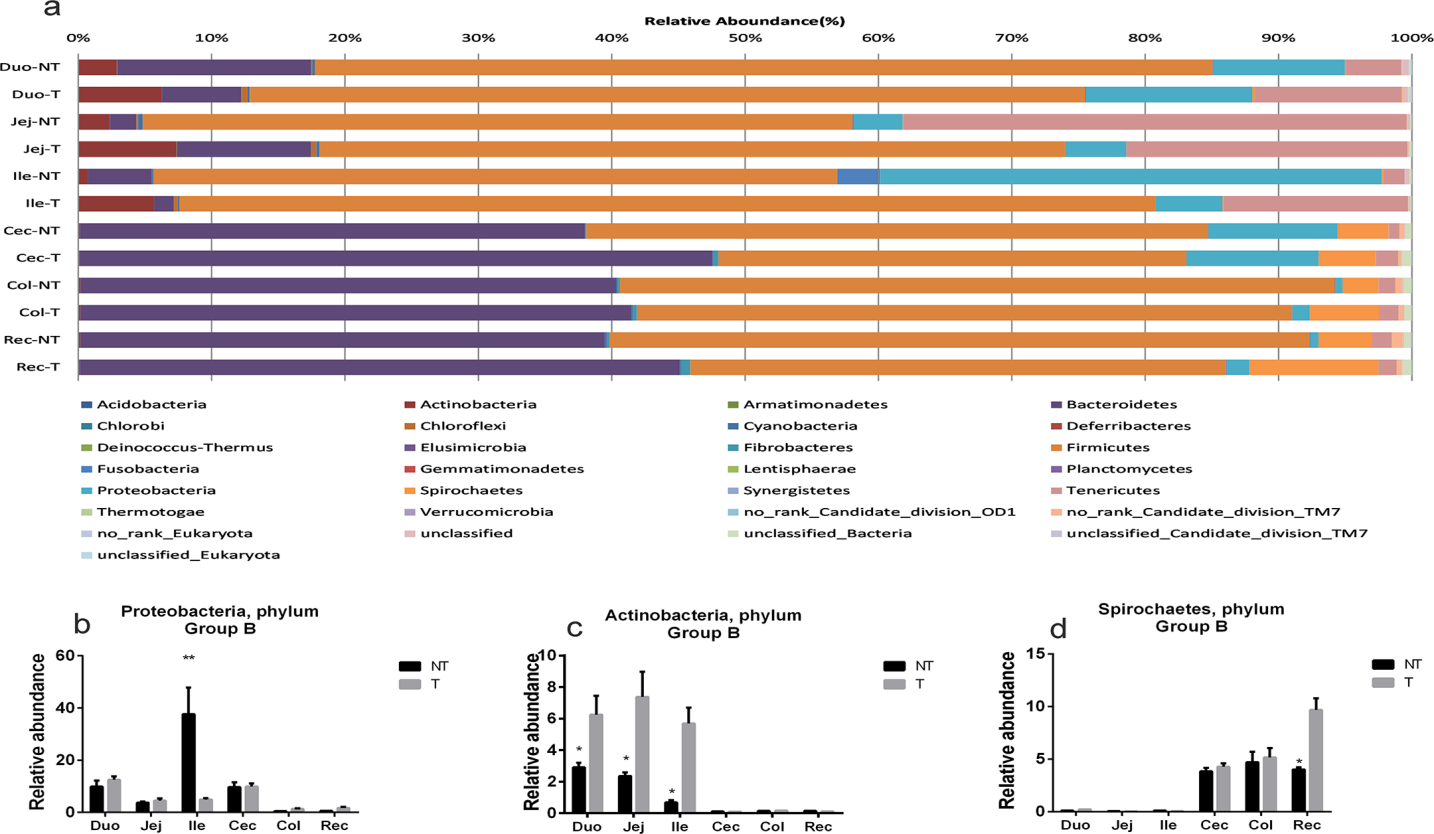
Comparative analysis of phyla in various intestinal sections collected from transgenic (T) and non-transgenic pigs (NT) in Group B: relative abundances of bacterial phyla (a) in fecal samples collected from various intestinal sections (duodenum, jejunum, ileum, cecum, colon, and rectum) of transgenic and non-transgenic pigs; *Proteobacteria* (b), *Actinobacteria* (c), and *Spirochaetes* (d) levels were significantly different in fecal samples collected from various intestinal sections between transgenic and non-transgenic pigs. *P<0.05; **P<0.01.

We noticed that there was a significant difference in the relative abundance of *Firmicutes* and *Bacteroidetes* at 0D transgenic and non-transgenic pigs in group A. Although the exact reason is not known, we speculate that this big difference could be due to the unintended effects on gut microbiota in transgenic pigs. Here we conducted a study on the unintended effects in Group B piglets. Statistical analysis of 29 phyla from non-transgenic and transgenic pigs in Group B indicated that the phyla *Firmicutes*, *Bacteroidetes*, *Tenericutes*, *Proteobacteria*, *Actinobacteria* and *Spirochaetes* are the most abundant, accounting for approximately 90% of total bacterial population (**Fig 5A**). In general, although no obvious difference in the above six phyla was observed for the same intestinal sections between transgenic and non-transgenic pigs, significant differences were noted for certain phyla in some sections of the intestinal tract (**S2 Table**). For example, the relative abundance of the phylum *Proteobacteria* in the ileum was significantly greater in non-transgenic pigs (37.6%) than in transgenic pigs (5.0%) (P-value = 0.0091) (**Fig 5B**). Because the relative abundance of *Proteobacteria* in non-transgenic pigs was similar to that previously reported[25], the significant decrease in the relative abundance of *Proteobacteria* observed in the ileum of transgenic pigs may indicate that unintended effects were induced by the *neo* transgene. The relative abundance of the phylum *Actinobacteria* in the duodenum, jejunum, and ileum was much lower in non-transgenic pigs than in transgenic pigs. For example, *Actinobacteria* accounted for 2.90%, 2.36%, and 0.68% of the total population in the duodenum, jejunum and ileum of non-transgenic pigs and 6.25%, 7.38%, and 5.69% of the total population in the duodenum, jejunum, and ileum of transgenic pigs (**Fig 5C**). The relative abundance of the phylum *Spirochaetes* in the rectum of non-transgenic pigs (4.0%) was much lower than that of transgenic pigs (9.67%, P-value = 0.047, see **Fig 5D**).

**Fig 6:**
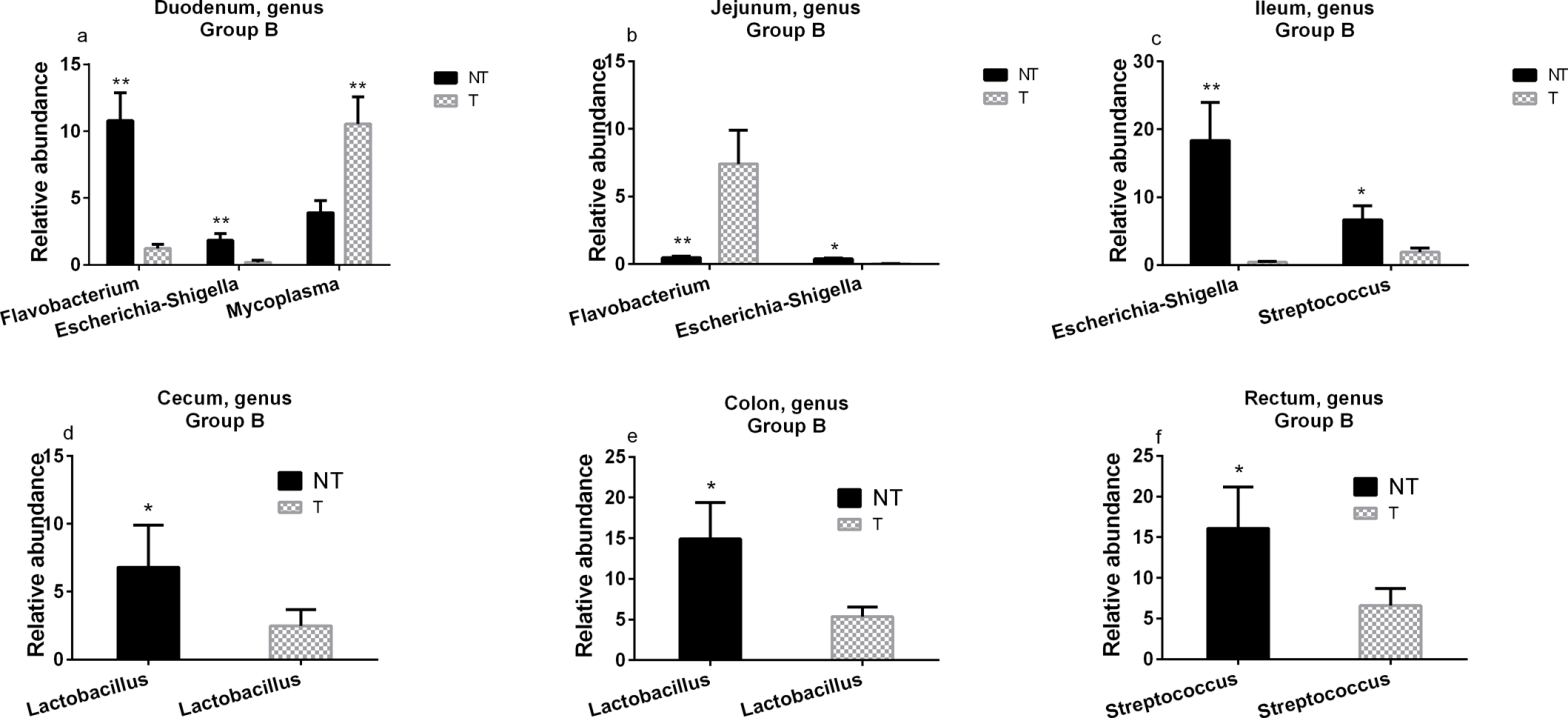
Comparative analysis of genera in various intestinal sections collected from transgenic and non-transgenic pigs in Group B. Significant differences between transgenic (T) and non-transgenic (NT) pigs were observed in changes in the relative abundance at the genus level in fecal samples from the duodenum (a), jejunum (b), ileum (c), cecum (d), colon (e), and rectum (f). *P<0.05; **P<0.01.

Analysis at the genus level was performed for fecal samples collected from different intestinal sections in all Group B pigs (**S5 Fig**). Further statistical analysis of 16 genera (accounting for 79–89% of the total microbiota) indicated that although no overall difference was noted in various sections of the intestinal tract between non-transgenic and transgenic pigs, significant differences were observed for some sections and certain genera (**S3 Table**). The relative abundances of *Flavobacterium* and *Escherichia-Shigella- Hafnia* in duodenum were much higher in non-transgenic pigs than in transgenic pigs (10.78% and 1.83% vs 1.24% and 0.20%, P-value = 0.0085 and P-value = 0.0081), while the relative abundance of *Mycoplasma* was much lower in non-transgenic pigs than in transgenic pigs (3.9% vs 10.55%, P-value = 0.0092) (**Fig 6A**). The relative abundance of *Flavobacterium* in the jejunum was much lower in non-transgenic pigs than in transgenic pigs (0.48% vs 7.41%, P-value = 0.008), whereas the relative abundance of *Escherichia-Shigella-Hafnia* in the jejunum was much greater in non-transgenic pigs than in transgenic pigs (0.40% vs 0.025%, P-value = 0.04) (**Fig 6B**). The relative abundances of *Streptococcus* and *Escherichia-Shigella-Hafnia* in the ileum were much higher in non-transgenic pigs than in transgenic pigs (6.66% and 18.35% vs 1.89% and 0.46%, P-value = 0.045 and P-value = 0.005) (**Fig 6C**). The relative abundance of *Lactobacillus* in the cecum and colon was much higher in non-transgenic pigs than in transgenic pigs (6.81% and 14.90 vs 2.48 and 5.35%, P-value = 0.046 vs 0.042) (**Fig 6D and 6E**). The relative abundance of *Streptococcus* in the rectum was much higher in non-transgenic pigs than in transgenic pigs (16.0% vs 6.6%, P-value = 0.039) (**Fig 6F**).

The overall distribution of bacterial populations in large intestine and small intestine was analyzed using Principal Coordinate Analysis (PCoA). Results indicated that, in both transgenic and non-transgenic pigs, bacterial communities clustered closely to one another in the large intestine, but did not cluster in the small intestine. This is consistent with previous observations[26]. At the same section of intestine, bacterial communities in non-transgenic pigs clustered closely to one another while those of transgenic pigs did not (**S6 Fig**).

## Discussion

The transgenic pigs used in this study contain *neo* and enhanced Green Fluorescent Protein (*eGFP*) transgenes. EGFP is a protein from the jellyfish *Aequorea victoria* and is widely used as marker for studying gene regulation in transgenic research field[27]. Although many reports have shown that *eGFP* has no toxicity to cells, there are no published data or reports on the effect of the *eGFP* transgene on the gut microbiota. Because *eGFP* is not a resistance-related protein, it is reasonable to believe that eGFP would not have any impact on the gut microbiota in animals.

Horizontal gene transfer refers to the transfer of genes between different species. It has been reported that horizontal gene transfer may occur inside the intestinal system in transgenic animals[7, 28]. Our lab previously showed that no horizontal gene transfers from transgenic pigs to gut microbiota was detected using PCR method for a drug non-resistance gene (*sFat-1*)[15]. Thus far, there is no report or evidence showing that the antibiotic resistance gene can transfer from the host’s intestinal tract to gut microbiota via bacterial integrins[29]. The *neo* gene is routinely used in basic research and in the production of genetically engineered animals; thus, it is necessary to detect or monitor the horizontal transfer of *neo* gene. Because the porcine gut microbiota does not contain any regulatory sequence element such as the CMV or SV40 promoters found in the *neo* transgene plasmid (see **Fig 1A**), we employed specific PCR primers to detect SV40 (which controls *neo* transgene expression) to evaluate whether horizontal gene transfer had occurred. Our data demonstrate that it is unlikely that there was any horizontal gene transfer in transgenic pigs by using our PCR assay method.

In Group A animals, there exists a difference in the magnitude of changes in *Firmicutes* and *Bacteroidetes* between transgenic and non-transgenic pigs during the period of neomycin feeding, but the overall pattern of changes in populations of *Firmicutes* and *Bacteroidetes* is almost the same between transgenic and non-transgenic pigs. However, there is a significant difference in overall pattern of changes in populations of *Proteobacteria* between transgenic and non-transgenic pigs during the period of neomycin feeding (see **Fig 3D**). In the late stages post antibiotic (neomycin) feeding, *Proteobacteria* recovered to the same level as at day 0 in transgenic pigs but stayed low in non-transgenic pigs. These significant differences in magnitude and pattern between transgenic and non-transgenic pigs demonstrated that the presence of secreted NPT II in guts of transgenic pigs can inhibit neomycin activity and thus could protect phylum *Proteobacteria* against neomycin to a certain degree, resulting a rapid recovery post neomycin feeding. On the other hand, more *Proteobacteria* could be killed by neomycin in non-transgenic pigs and thus resulted in a slower recovery post neomycin feeding.

In Group B animals, we noticed that there exist some differences between transgenic and non-transgenic pigs for some gut bacteria. We speculate that this may be due to the unintended effects of *neo* transgene on gut microbiota in transgenic pigs. After analysis of bacteria at genus level, it was found that the unintended effects could be either positive or negative. For example, in Group B pigs at 3 months, transgenic pigs apparently have a drastically reduced amount of lactobacilli compared with non-transgenic pigs. In addition, the relative abundance of *Mycoplasma* was much higher in transgenic pigs than in non-transgenic pigs, implying that individual transgenic pigs could be more susceptible to *Mycoplasma* infection than non-transgenic pigs. Since both non-transgenic and transgenic pigs were housed together, we speculate that individual non-transgenic pigs may get infected with *Mycoplasma* by means of direct contacts between transgenic pigs and non-transgenic pigs through saliva and feces. Although Mycoplasma was detected, all animals (both non-transgenic and transgenic pigs) in our study did not show any sign of fever, increased saliva flow, and frothy discharge from nostrils. It is well known that there are a variety of *Mycoplasma* species, the sequencing data from our current study only matched *Mycoplasma* at genus level, and thus we cannot determine specific species of *Mycoplasma* in this experiment. However, we are going to conduct more experiments related to identification of *Mycoplasma* species in the future (see **Fig 6A, 6D and 6E**). These data indicate that *neo* transgene expression could have potential negative effects on animal health by decreasing the relative abundance of beneficial bacteria such as *Lactobacillus* in the intestines. It had been previously reported that the increase in *Escherichia-Shigella-Hafnia* is a key factor to cause diarrhea in pigs[30]. Our data showed that the relative abundance of *Escherichia-Shigella-Hafnia* was much lower in transgenic pigs than in non-transgenic pigs (see **Fig 6A–6C**), which may imply that this decrease in *Escherichia-Shigella-Hafnia* could be positive to transgenic pigs.

In summary, our current study showed that horizontal gene transfer is unlikely to occur from transgenic pigs to the gut microbiota. The significant differences of changes in the patterns and magnitude of phylum *Proteobacteria* between transgenic and non-transgenic pigs during neomycin feeding demonstrate that the presence of NPT II offered some protection of certain gut bacterial populations against neomycin via the inhibition of neomycin activity by NPT II per se. *Neo* transgene expression in transgenic pigs also generated unintended effects on the balance of gut microbiota, resulting not only in changes in the relative abundance of some bacteria at the phylum and genus levels in some intestinal sections but also in a decrease in the levels of some beneficial bacteria such as *Lactobacillus* or a decrease in the levels of potentially harmful bacteria such as *Escherichia-Shigella-Hafnia*. Thus, it is clear that the *neo* transgene has various effects on the gut microbiota. Although the mechanism by which the *neo* transgene exerts its effect on the intestinal microbiota requires further investigation, our study clearly raises a scientific issue and cautions over the wide use of the *neo* transgene in transgenic animal.

## Methods

### Experimental Design and Sample Collection

All transgenic pigs were produced by BGI Tech using handmade somatic cell cloning. The *neo* gene was incorporated into porcine cells using the PEZ1.1 plasmid, shown in **Fig 1A**. All piglets in Group A come from the same mother (sow 262), and all piglets in Group B also come from the same mother (sow 264). Of these piglets, 8 (4 transgenic and 4 non-transgenic) from sow 262 were assigned to Group A, and 10 (5 transgenic and 5 non-transgenic) from sow 264 were assigned to Group B. All piglets were weaned at 1 month and were then fed a standard diet, except when otherwise indicated. All animal care procedures were approved by the Institutional Animal Care and Use Committee (IACUC) of the Chinese Academy of Agricultural Sciences prior to initiation of the experiment. All experiments were performed in accordance with the approved guidelines. All piglets were maintained in two environmentally controlled pigpens. Group A pigs were fed a standard diet supplemented with neomycin sulfate (100 g neomycin sulfate added to 500 kg standard diet) at day 45 after birth; the feeding was continued daily for a total of seven days, and the pigs were then fed a standard diet until the end of the experiment (3 months). Group B pigs were fed a standard diet throughout the entire study period. To keep the pigpens clean, they were cleaned with water every hour. The drinking water for all experimental pigs was autoclaved prior to feeding. At the indicated time points (at days 0, 8, 23 and 45 post neomycin feeding), each pig in Group A was injected with 4 mL of glycerol into the rectum to collect fecal samples. At the end of 3 months, each pig in Group B was euthanized by method of electrocution which was approved by the IACUC to collect samples from the duodenum, jejunum, ileum, cecum, colon, and rectum. All samples were stored at -80°C until DNA extraction.

### Multiplex PCR

Multiplex PCR was employed to detect horizontal gene transfer from animal tissues/cells to the gut microbiota. Primers P1 (2018) and P2 (1684) were designed to amplify the *neo* gene, Primers P3 (1908) and P4 (2753) were designed to amplify the SV40 promoter and the *neo* gene, Primers P5 and P6 were designed to detect the porcine beta-actin gene, and Primers P7 and P8 were used to amplify bacterial 16S rDNA. The primer sequences are listed below: P3: 5′-*AGACAGGATGAGGATCGTTTCG*-3′; P4: 5′-*CGGTCATTTCGAACCCCAGA*-3′; P5: 5′-*GCCAACCGTGAGAAGATG*-3′; P6: 5′-*TGCAAGGAACACGGCTAA*-3′; P7:5′-*CGCCCGGGGCGCGCCCCGGGCGGGGCGGGGGCACGGGGGAACGCGAAGAACCTTAC*-3′; and P8: 5′-*CGGTGTGTACAAGACCC*-3′. The multiplex PCR reaction comprised the following components in a 20 μL volume: 2 μL of 10× PCR buffer; 1.0 μL of dNTPs; 0.6 μL of primers (P1-P8, 10 pmol/μL); 100 ng of template DNA; and 0.2 μL (1 U) of rTaq DNA polymerase. Purified water was added to a final volume of 20 μL. The PCR reaction conditions were as follows: denaturation at 95°C for 5 min; 30 cycles of denaturation at 95°C for 30 s, temperature gradient annealing at 60°C for 30 s, and extension at 72°C for 50 s; and extension at 72°C for 5 min. Three μL of each PCR product was then run on a 2% agarose gel.

### PCR

Forward and reverse primers to detect the *neo* gene were designed as P1 (2018) and P2 (2684), as follows: P1 sequence: 5′-*CAAGATGGATTGCACGCAGG*-3′; and P2 sequence: 5′-*GGTAGCCAACGCTATGTCCT*-3′. The total PCR reaction volume was using 20 μL, containing 2 μL 10× amplification buffer, 2 μL dNTP mixture (200 μmol/L each), 0.5 μL P1 (10 pmol) and 0.5 μL P2 (10 pmol), 100 ng template DNA, and 0.2μL Taq DNA polymerase, 1.5 μL Mg^2+^ (1.5 mmol/L); purified water was added to a total volume of 20μL. The PCR reaction conditions were as follows denaturation at 95°C for 5 min; 30 cycles of denaturation at 95°C for 30 s, temperature gradient annealing at 60°C for 30 s, and extension at 72°C for 45 s; and extension at 72°C for 5 min. Three μL of each PCR product was then analyzed by 2% agarose gel electrophoresis (120 V voltage for 30 min).

### Southern Blot

Total genomic DNA was extracted from the ear of each experimental pig. Ten micrograms of genomic DNA was digested by SalⅠand Hind Ⅲ. After being separated by 1% agarose gel electrophoresis, DNA was blotted onto a nylon membrane and then probed with *neo* cDNA using a DIG High Prime DNA Labeling and Detection Starter Kit I (Roche, Germany).

### Western Blot

Intestinal tissue extracts were separated by SDS-PAGE (4–12% gradient gel) and transferred to a nitrocellulose membrane. The membrane was blocked with 5% nonfat milk in 50 mM Tris-HCl (pH 7.4), 100 mM NaCl, and 0.1% Tween-20 (TBST) and then incubated with purified mouse anti-NPTⅡ antibodies (0.5 mg/ml) in 5% nonfat milk in TBST at room temperature for 3 h. The blot was washed (4 times, 10 min each) with TBST and further incubated with a 1:2,500 dilution of horseradish peroxidase (HRP)-conjugated goat anti-mouse IgG antibodies (HRP; DAKO, Carpinteria, CA) for 1 h at room temperature. The membrane was then washed as described above and detected using an enhanced chemiluminescence kit according to the manufacturer’s protocol.

### ELISA

All fecal samples were centrifuged at 3000 rpm for 10 min to collect the supernatants. To each well, 100 μL of supernatant was added and then incubated at room temperature for 2 h and washed 7 times with 300 μL of washing buffer; the prepared enzyme conjugate was then added to each well, incubated at room temperature for 2 h and washed 8 times with 300 μL washing buffer. The TMB substrate solution was added, and the OD was measured after incubation for 15 min at room temperature.

### DNA Extraction and Purification

Fecal samples (180 mg) were suspended in 1400 μL of ASL buffer, and genomic DNA was extracted using a QIAamp DNA Stool Mini Kit (Qiagen, Germany), following the manufacturer’s instructions with slight modifications. Sterile zirconia beads were added to each sample. For each sample, DNA was extracted in duplicate to avoid bias, and the extracts from the same sample were pooled as one sample. The DNA purity and concentration were analyzed spectrophotometrically using an e-Spect ES-2 (Malcom, Japan). The extracted DNA was stored at -20C until use.

### PCR Amplification, Amplicon Quantitation, Pooling, and Pyrosequencing

A region of 526 bp in the 16S rRNA gene encoding the V1–V3 region was selected to construct a gut microbiota library through tag pyrosequencing. Primers 27F and 533R, containing the A and B sequencing adaptors (454 Life Sciences), were used to amplify this region. The forward primer (B-27F) was 5’-*CCTATCCCCTGTGTGCCTTGGCAGTCTCAGAGAGTTTGATCCTGGCTCAG*-3’; the sequence of the B adaptor is shown in italics and underlined. The reverse primer (A-533R) was 5’-*CCATCTCATCCCTGCGTGTCTCCGACTCAGNNNNNNNNNNTTACCGCGGCTGCTGGCAC*-3’; the sequence of the A adaptor is shown in italics and underlined, and the Ns represent an eight-base sample-specific barcode sequence. The length of the amplicon was 596bp. PCR was carried out in triplicate 50 μL reactions containing 0.6 mM primer, 5 ng of template DNA, 1x PCR reaction buffer, and 2.5 U Pfu DNA Polymerase (MBI, Fermentas, USA). The following amplification program was used: an initial denaturation step at 94°C for 4 min, followed by 25 cycles of 94°C for 30 s (denaturation), 55°C for 30 s (annealing) and 72°C for 30 s (extension), and a final extension step at 72°C for 10 min. Negative control assays were also performed. PCR products were visualized on agarose gels (2% in TBE buffer) containing ethidium bromide and were purified with a DNA gel extraction kit (Axygen, China). The DNA concentration of each PCR product was determined using a Quant-iT PicoGreen double-stranded DNA assay (Invitrogen). Amplicons from each PCR were then pooled in equimolar ratios and subjected to emulsion PCR to generate amplicon libraries per the instructions from 454 Life Sciences. Amplicon pyrosequencing was performed from the A-end using a 454/Roche A sequencing primer kit on a Roche Genome Sequencer GS FLX Titanium platform at Majorbio Bio-Pharm Technology Co., Ltd., Shanghai, China.

### Data analysis

From the result of rarefaction analysis (**S7 Fig**), we confirmed that the raw data of sequencing has high degree of coverage required (**S4 and S5 Tables**). By calculating the Shannon index of the raw data from each sample (**S8 Fig**), we confirmed that the high degree of sequencing consistence was achieved. Pyrosequencing reads with more than one ambiguous nucleotide or within correct barcodes or primers were removed and excluded from further analysis. Sets of sequences with 97% identity were defined as Operational Taxonomic Units (OTUs). OTUs were assigned to a taxonomy using the Ribosomal Database Project (RDP) Naive Bayes classifier[31]. Representative sequences from each cluster were aligned with the PyNAST aligner[32] to the Greengenes core set in QIIME[33]. A phylogenic tree was constructed within QIIME using FastTree [34]. Rarefaction curves, alpha diversity, and beta diversity calculations were also performed using QIIME. The Shannon diversity index (SI) was estimated to evaluate the ecological diversity of the gut microbiota from each sample. The SI is a quantitative measure that reflects how many different types (such as species) exist in a dataset and simultaneously accounts for how evenly the basic entities (such as individuals) are distributed among those types. The value of a diversity index increases as the number of types increases and as evenness increases. However, the interpretation is hindered by uncertain species definitions and the lack of a statistical framework for comparing values. The OTU network was constructed by QIIME and visualized using Cytoscape to map the gut microbial community composition and structure onto the porcine GI tract, thereby complementing phylogeny-based microbial community comparisons. These analyses were used to bin 16S rRNA V1-V3 gene sequences into OTUs and to display microbial genera partitioning across pig GI tracts. The OTUs and individual samples were designated as nodes in a bipartite network, in which OTUs were connected to the samples in which their sequences were found. A spring-embedded algorithm was used to cluster the OTUs and samples. A Principal Coordinate Analysis (PCoA) was performed based on weighted UniFrac distance.

### Statistical analysis

Quality control and normalization were performed for all raw sequencing data. Changes in bacterial abundance were then compared using repeated measures ANOVA with Mann-Whitney U test. Relationships between sequences as well as diversity and coverage were examined by Spearman correlation. Statistical analyses were performed using GraphPad Prism (version 5.0.1, GraphPad Software Inc., San Diego, CA, USA).

## Supporting Information

S1 Fig Different patterns and/or magnitudes in changes in the phylum level between transgenic and non-transgenic pigs post neomycin feeding in Group A: comparison of the relative abundance of bacterial phyla (a) in fecal samples collected from the rectums of transgenic and non-transgenic pigs at various time points (0D, 8D, 23D and 45D) post neomycin feeding; comparison of changes in the levels of *Firmicutes* (b), *Bacteroidetes* (c), and *Proteobacteria* (d) at various time points (0D, 8D, 23 and 45D) post neomycin feeding between transgenic (T) and non-transgenic (NT) pigs in Group A. 0D vs 8D: $: P<0.05, $ $: P<0.01; 8D vs 23D: #: P<0.05; 23D vs 45D: &: P<0.05.
(TIF)

S2 Fig Comparison of the relative abundance of bacterial genera in fecal samples collected from the rectums of transgenic and non-transgenic pigs at various time points (0D, 8D, 23D and 45D) post neomycin feeding. (TIF)

S3 Fig Different patterns and/or magnitudes in the changes in genus levels between transgenic and non-transgenic pigs post neomycin feedings in Group A: comparison of changes in the levels of *Lactobacillus* (a) and genus *Prevotella* (b) at various time points (0D, 8D, 23 and 45D) post neomycin feeding between transgenic (T) and non-transgenic (NT) pigs. 0D vs 8D: $: P<0.05, $ $: P<0.01; 8D vs 23D: #: P<0.05; 23D vs 45D: &: P<0.05.
(TIF)

S4 Fig The principal coordinate analysis (PCoA) of each sample in Group A. NT: non-transgenic pigs; T: transgenic pigs.
(TIF)

S5 Fig Relative abundances of bacterial genera in fecal samples collected from various intestinal sections (duodenum, jejunum, ileum, cecum, colon, and rectum) of transgenic (T) and non-transgenic (NT) pigs in Group B. (TIF)

S6 Fig The principal coordinate analysis (PCoA) of each sample in Group B. NT: non-transgenic pigs; T: transgenic pigs.
(TIF)

S7 Fig Rarefaction analysis of different fecal samples. Rarefaction curves of OTUs clustered at 97% sequence identity across different fecal samples.
(TIF)

S8 Fig Shannon index in the fecal samples (Group A pigs) at various time points post antibiotic (neomycin) feeding and in fecal samples from various intestinal sections (Group B pigs). The Shannon index was estimated to evaluate the ecological diversity of microbiota from each sample. The Shannon index is relatively consistent for samples at various time points in Group A pigs. The Shannon index varies significantly for small intestinal samples in Group B pigs but is relatively consistent for large intestinal samples. Pigs 1–4, non-transgenic pigs in Group A; pigs 5–8, transgenic pigs in Group A; pigs 9–13, non-transgenic pigs in Group B; and pigs 14–18, transgenic pigs in Group B.S1 Table Analysis of the changes at the genus level between transgenic and non-transgenic pigs from Group A at various time points post neomycin feeding.
(TIF)

S1 Table Analysis of the changes at the genus level between transgenic and non-transgenic pigs from Group A at various time points post neomycin feeding. (DOCX)

S2 Table Comparative analysis of the relative abundance of six phyla in different intestinal sections from transgenic and non-transgenic pigs in Group B. (DOCX)

S3 Table Comparative analysis of the relative abundance of bacterial genera in different intestinal sections from transgenic and non-transgenic pigs in Group B. (DOCX)

S4 Table Overview of pyrosequencing results for fecal samples in Group A collected at various time points post antibiotic feeding. (DOCX)

S5 Table Overview of pyrosequencing results for fecal samples in Group B collected from different intestinal sections. (DOCX)
