# Supplementary figures and images for: Safety Evaluation of Neo Transgenic Pigs by Studying Changes in Gut Microbiota Using High-Throughput Sequencing Technology
Source: PLoS One. 2016 Mar 11;11(3):e0150937. doi: 10.1371/journal.pone.0150937 (PMC4788350; doi:10.1371/journal.pone.0150937)

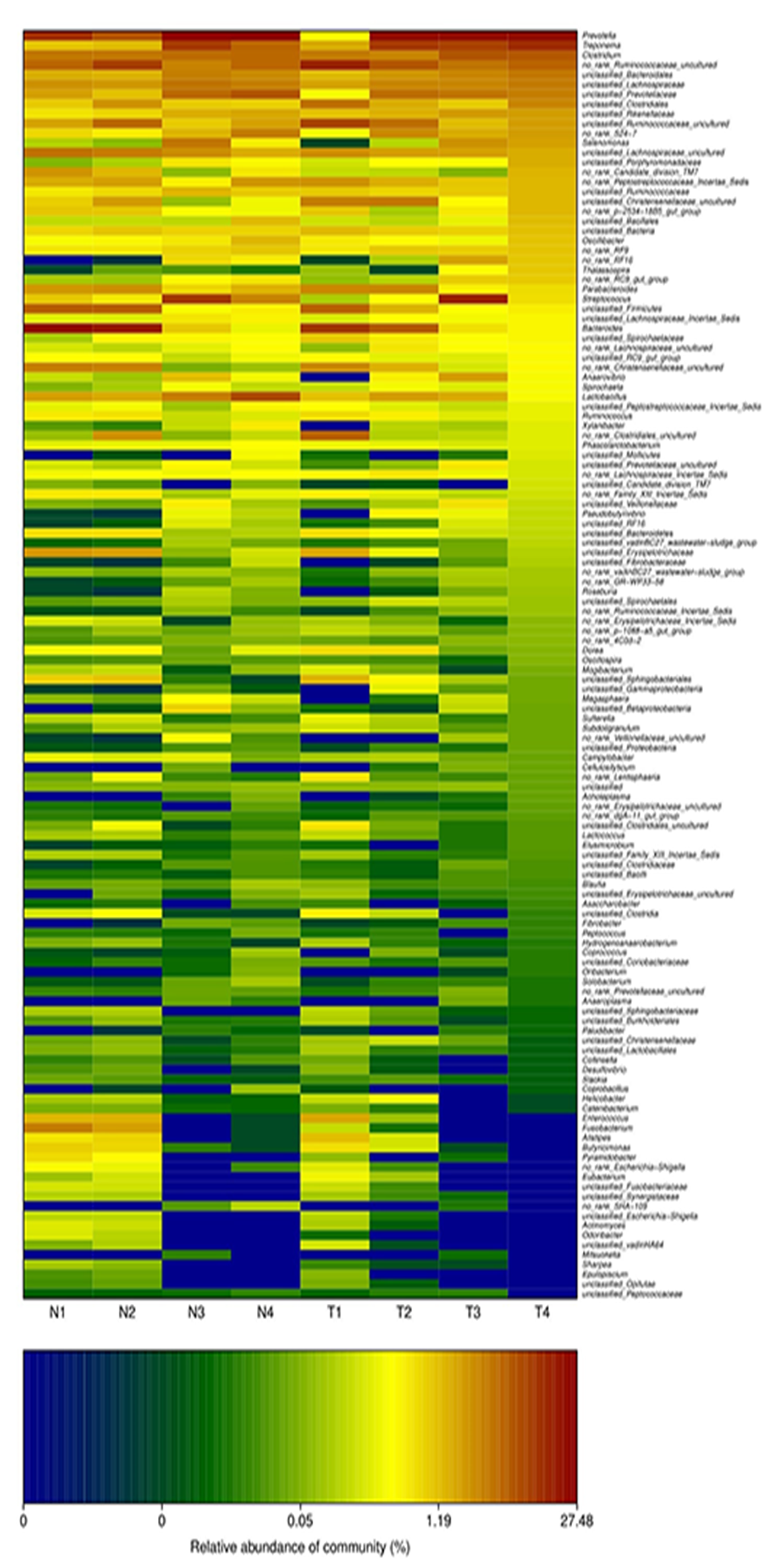

Supplement: S2 Fig — (TIF) [file pone.0150937.s002.tif]

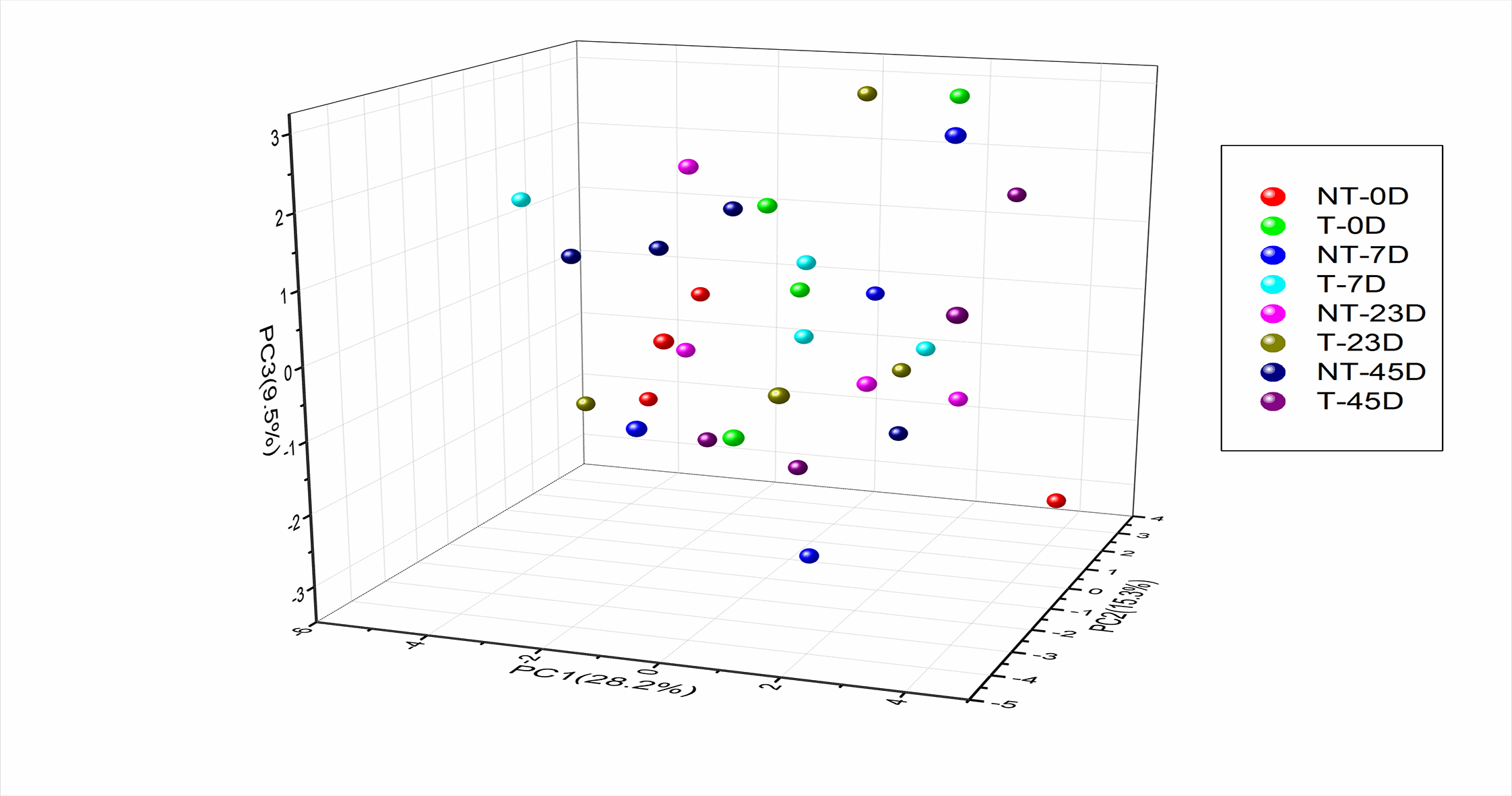

Supplement: S4 Fig — NT: non-transgenic pigs; T: transgenic pigs. (TIF) [file pone.0150937.s004.tif]

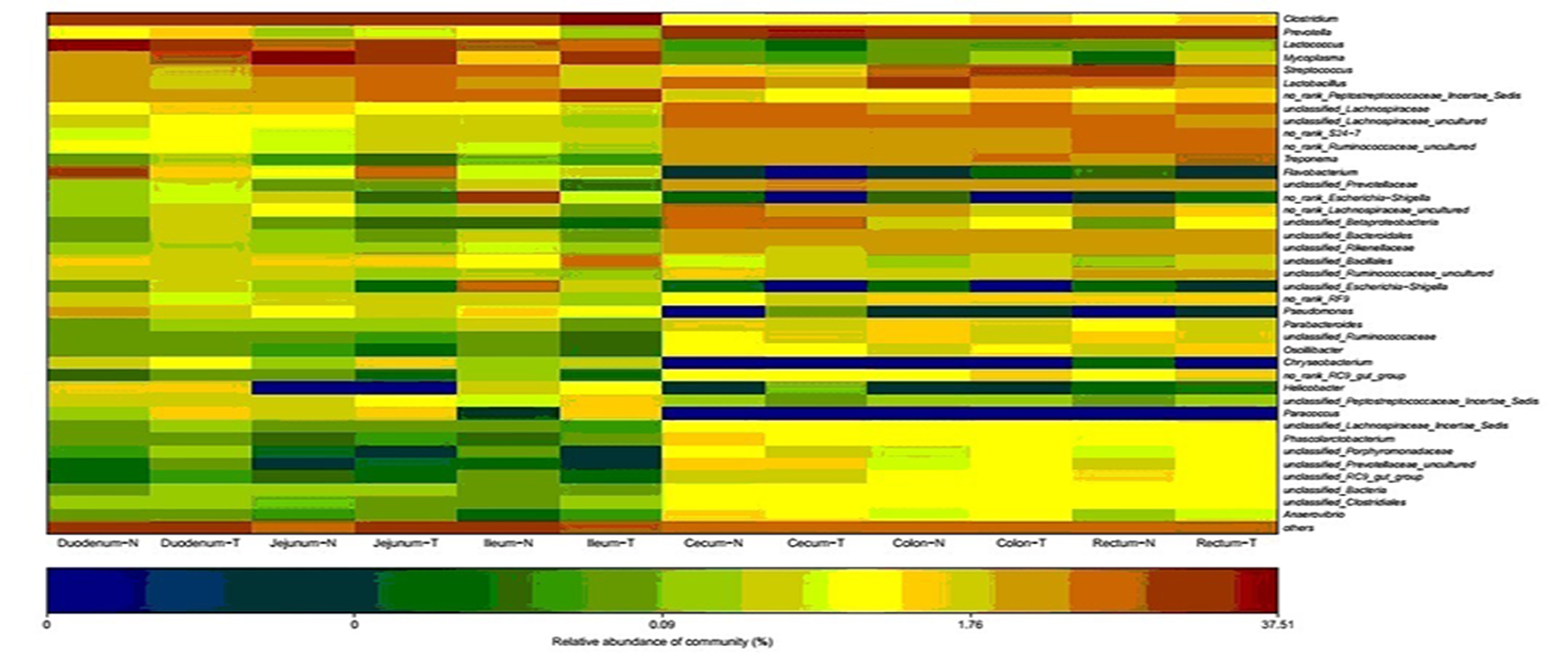

Supplement: S5 Fig — (TIF) [file pone.0150937.s005.tif]

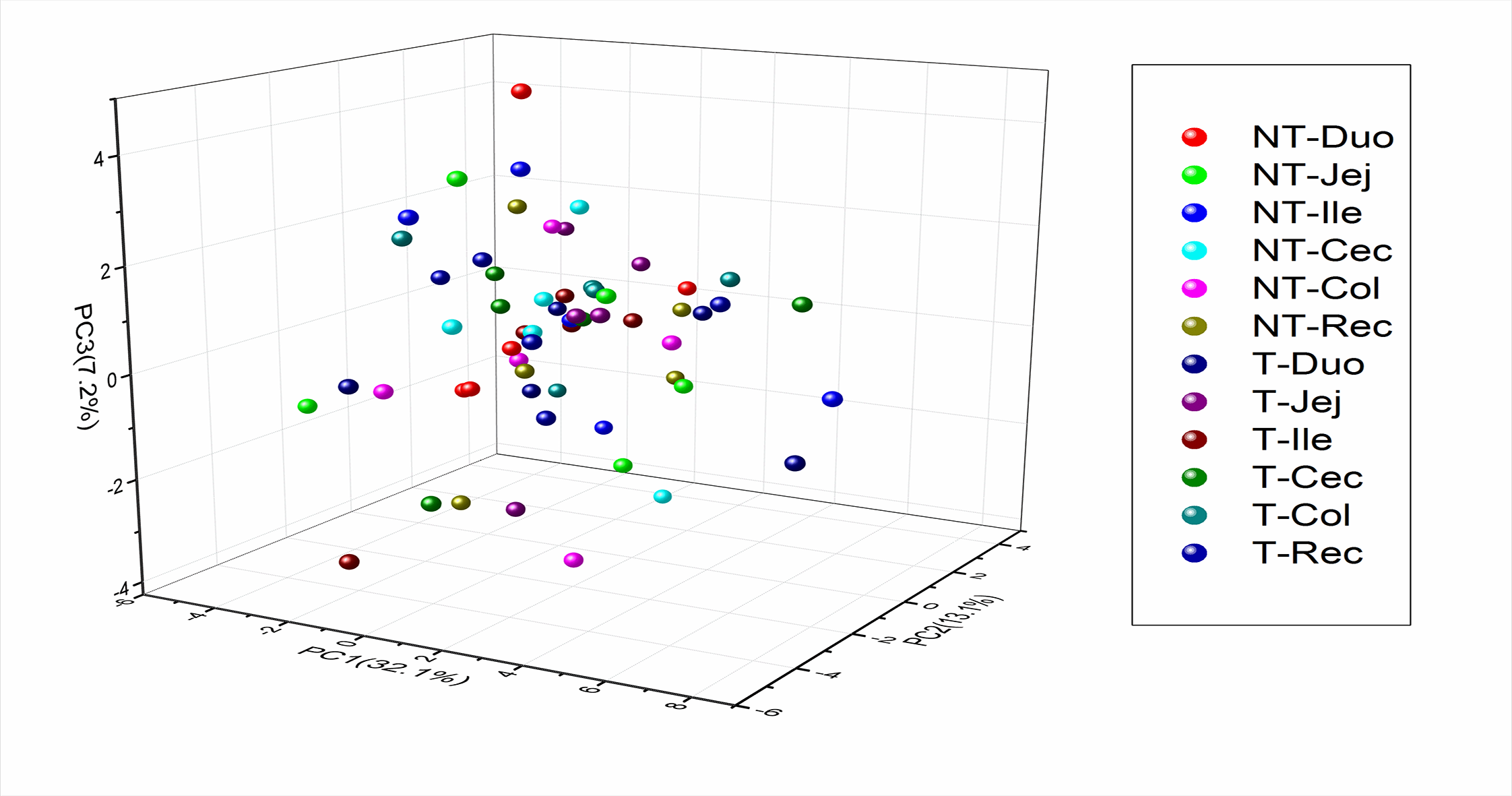

Supplement: S6 Fig — NT: non-transgenic pigs; T: transgenic pigs. (TIF) [file pone.0150937.s006.tif]

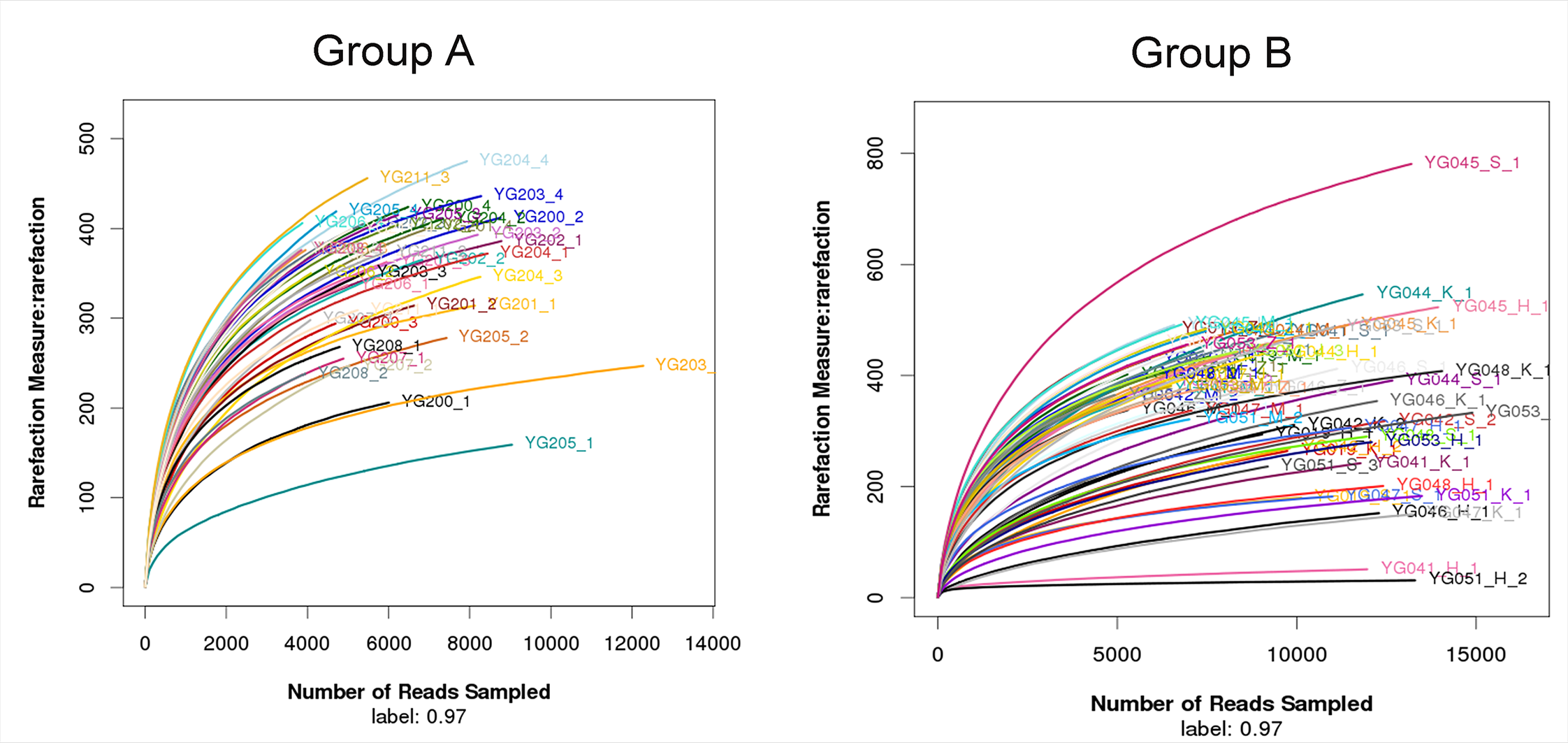

Supplement: S7 Fig — Rarefaction curves of OTUs clustered at 97% sequence identity across different fecal samples. (TIF) [file pone.0150937.s007.tif]

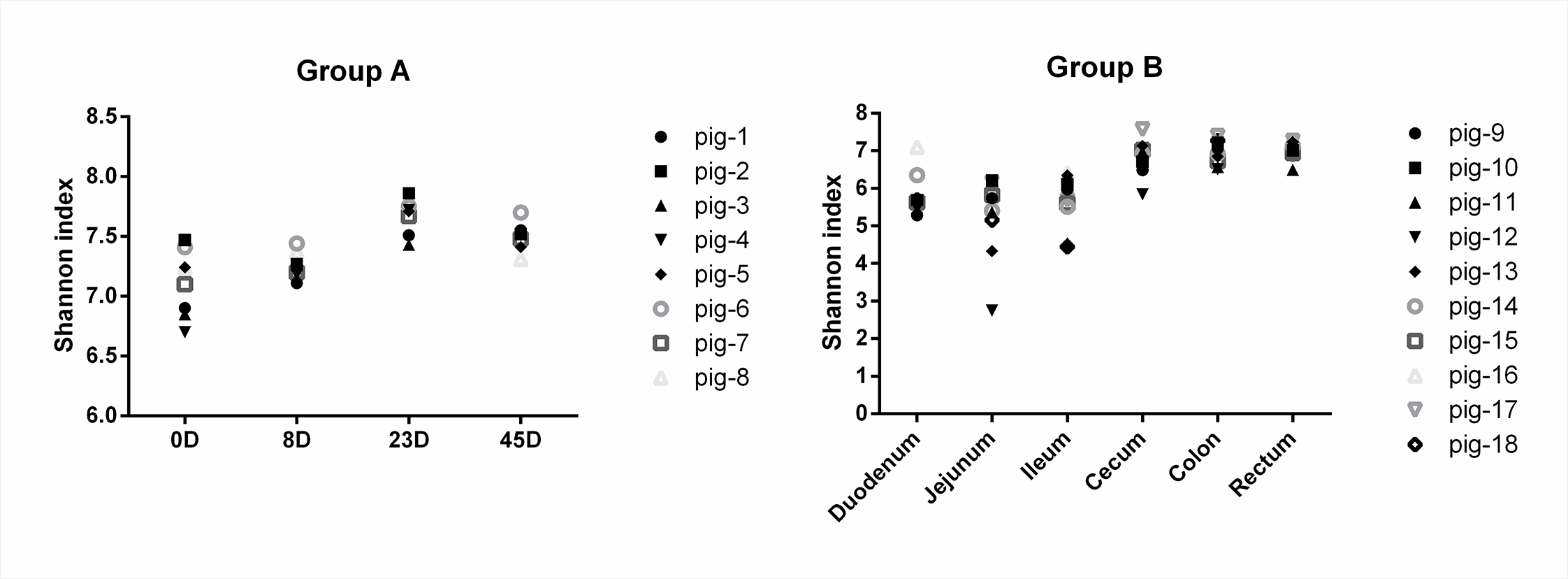

Supplement: S8 Fig — Shannon index in the fecal samples (Group A pigs) at various time points post antibiotic (neomycin) feeding and in fecal samples from various intestinal sections (Group B pigs). The Shannon index was estimated to evaluate the ecological diversity of microbiota from each sample. The Shannon index is relatively consistent for samples at various time points in Group A pigs. The Shannon index varies significantly for small intestinal samples in Group B pigs but is relatively consistent for large intestinal samples. Pigs 1–4, non-transgenic pigs in Group A; pigs 5–8, transgenic pigs in Group A; pigs 9–13, non-transgenic pigs in Group B; and pigs 14–18, transgenic pigs in Group B.S1 Table Analysis of the changes at the genus level between transgenic and non-transgenic pigs from Group A at various time points post neomycin feeding. (TIF) [file pone.0150937.s008.tif]
