# Supplementary material for: Safety Evaluation of Neo Transgenic Pigs by Studying Changes in Gut Microbiota Using High-Throughput Sequencing Technology
Source: PLoS One. 2016 Mar 11;11(3):e0150937. doi: 10.1371/journal.pone.0150937 (PMC4788350; doi:10.1371/journal.pone.0150937)
Supplement: S1 Table — (DOCX) [file pone.0150937.s009.docx]

**S1 Table. Analysis of changes in genus level in fecal samples between transgenic and non-transgenic pigs from Group A at various time points post neomycin feedings**

| Name | | 0D-NT（%） | 8D-NT（%） | 23D-NT（%） | 45D-N（%） | 0D-T（%） | 8D-T（%） | 23D-T（%） | 45D-T（%） |
| --- | --- | --- | --- | --- | --- | --- | --- | --- | --- |
| Prevotella | | 12.09^$^ | 36.14^#^ | 24.96 | 27.45 | 0.01^&^ | 21.65 | 21.5 | 21.81 |
| Bacteroides | | 19.73^$^ | 2.73 | 3.46 | 2.93 | 10.06^$^ | 5.62 |  |  |
| no_rank_Ruminococcaceae_uncultured | | 4.98 | 3.82 | 3.32 | 5.16 | 8.5 | 6.63 | 4.19 | 2.14 |
| unclassified_Lachnospiraceae_uncultured | | 4.12 | 2.83 | 4.1 | 3.54 | 2.67 | 3.11 | 3.11 | 3.6 |
| Clostridium | | 3.93 | 1.51 | 4.68 | 5.1 | 3.69 | 3.3 | 7.04 | 6.33 |
| unclassified_Lachnospiraceae | | 2.56 | 2.47 | 2.79 | 1.55 | 1.84 | 2.05 | 1.96 | 1.68 |
| Lactobacillus | | 2^$^ | 0.01^#^ | 3.3^&^ | 7.35 | 0.01^$^ | 1.99 | 1.63^&^ | 0.01 |
| unclassified_Ruminococcaceae_uncultured | | 1.93 | 1.41 | 1.4 | 2 | 14.34 | 4.5 | 1.32 | 5.22 |
| unclassified_Prevotellaceae | | 1.98^$^ | 5.19 | 4.79 | 6.33 |  | 4.47 | 3.97 | 3.45 |
| no_rank_S24-7 | |  | 5.01 | 1.49 | 3.53 |  | 2.86 | 1.7 | 2.07 |
| Parabacteroides | | 2.3 | 2.14 |  | 1.48 | 2.9 | 2.97 |  |  |
| Bacteroidales | | 1.67 | 1.95 | 1 |  |  | 2.38 | 3.13 | 3.92 |
| Streptococcus |  | | 0.01 | 9.8 | 3.09 |  |  | 14.44 |  |
| Treponema |  | | 5.14 | 10.29 | 5.36 | 1.62 | 10.4 | 9.22 | 13.65 |
| Rikenellaceae |  | |  | 1.53 | 1.73 |  | 1.6 | 2.17 | 2.18 |
| Erysipelotrichaceae | | 2.4 | 1.93 |  |  | 1.83 |  |  |  |
| Anaerovibrio |  | | 2.23 | 1.22 |  |  |  | 2.03 |  |
| Christensenellaceae | | 3.19 |  |  |  | 3.53 |  |  |  |
| Candidate_division_TM7 | | 2.34 |  |  |  |  |  |  | 1.43 |
| Fusobacterium | | 3.58 |  |  |  |  |  |  |  |
| Lachnospiraceae |  | |  |  |  |  |  |  |  |
| Selenomonas |  | |  | 3.88 |  |  |  | 2.49 | 2.04 |

NT: non-transgenic pigs, T: transgenic pigs. 0D vs 8D: $: P<0.05, $$: P<0.01; 8D vs 23D: #: P<0.05; 23D vs 45D: &: P<0.05, by Mann-Whitney U test.

no_rank_S24-7 belongs to Bacteroridales.
