# Supplementary material for: Safety Evaluation of Neo Transgenic Pigs by Studying Changes in Gut Microbiota Using High-Throughput Sequencing Technology
Source: PLoS One. 2016 Mar 11;11(3):e0150937. doi: 10.1371/journal.pone.0150937 (PMC4788350; doi:10.1371/journal.pone.0150937)
Supplement: S2 Table — (DOCX) [file pone.0150937.s010.docx]

**S2 Table. Comparative analysis of relative abundance of six phyla in different intestinal sections from transgenic and non-transgenic pigs in Group B**

| Sample ID | Firmicutes (%) | Bacteroidetes (%) | Proteobacteria (%) | Spirochaetes (%) | Actinobacteria (%) | Tenericutes (%) |
| --- | --- | --- | --- | --- | --- | --- |
| Duo-NT | 67.29 | 14.56* | 9.85 | 0.097 | 2.90 | 4.14 |
| Duo-T | 62.60 | 5.95 | 12.48 | 0.20 | 6.25 | 10.99 |
| Jej-NT | 53.23 | 3.69 | 3.70 | 0.042 | 2.35 | 37.74 |
| Jej-T | 55.91 | 10.05* | 4.54 | 0.023 | 7.38 | 21.04 |
| Ile-NT | 51.23 | 4.79 | 37.61** | 0.097 | 0.68 | 1.60 |
| Ile-T | 73.21* | 1.46 | 4.98 | 0.049 | 5.69 | 13.87 |
| Cec-NT | 46.63 | 37.84 | 9.69 | 3.83 | 0.01 | 0.80 |
| Cec-T | 35.08 | 47.42 | 9.91 | 4.25 | 0.10 | 1.67 |
| Col-NT | 53.60 | 40.16 | 0.52 | 2.68 | 0.14 | 1.26 |
| Col-T | 49.12 | 41.31 | 1.31 | 5.15 | 0.15 | 1.49 |
| Rec-NT | 52.50 | 39.32 | 0.61 | 4.00 | 0.14 | 1.47 |
| Rec-T | 40.12 | 44.98 | 1.72 | 9.67 | 0.11 | 1.33 |

NT: non-transgenic pigs, T: transgenic pigs, *P<0.05, **P<0.01, by Mann-Whitney U test, significantly different for intestinal segment and animal type.
