# Supplementary material for: Safety Evaluation of Neo Transgenic Pigs by Studying Changes in Gut Microbiota Using High-Throughput Sequencing Technology
Source: PLoS One. 2016 Mar 11;11(3):e0150937. doi: 10.1371/journal.pone.0150937 (PMC4788350; doi:10.1371/journal.pone.0150937)
Supplement: S3 Table — (DOCX) [file pone.0150937.s011.docx]

**S3 Table. Comparative analysis of relative abundance of bacterial genus in different intestinal sections from transgenic and non-transgenic pigs in Group B**

|  | Small intestine | | | | | |  | Large intestine | | | | | | |
| --- | --- | --- | --- | --- | --- | --- | --- | --- | --- | --- | --- | --- | --- | --- |
| Name | Duo-NT | Duo-T | Jej-NT | Jej-T | Ile-NT | Ile-T | Name | Cec-NT | Cec-T | Col-NT | Col-T | Rec-NT | Rec-T | |
| Clostridium | 21.25 | 20.91 | 21.16 | 20.78 | 19.79 | 37.07 | Prevotella | 16.97 | 25.54 | 19.47 | 20.72 | 17.14 | 21.78 |  |
| Lactococcus | 29.87 | 23.58 | 9.64 | 11.53 | 10.27 | 7.26 | Streptococcus |  |  | 9.48 | 10.84 | 15.92 | 6.64* |  |
| Streptococcus | 3.37 | 2.22 | 5.73 | 5.7 | 6.66 | 1.89* | Lachnospiraceae | 7.88 | 6.39 | 5.18 | 5.87 | 5.75 | 5.36 |  |
| Peptostreptococcaceae | 3.32 | 3.93 | 4.72 | 5.9 | 5.8 | 1.45 | no_rank_Lachnospiraceae_uncultured | 7.58 | 5.96 | 4.14 | 5.22 | 4.15 | 1.25 |  |
| Lactobacillus | 2.83 | 2 | 4.46 | 5.42 | 3.22 | 2.02 | Lactobacillus | 6.81 | 2.63 | 14.89 | 5.93 | 5.16 | 2.6 |  |
| Pseudomonas | 2.51 | 1.88 | 0.87 |  | 0.99 | 0.54 | no_rank_Lachnospiraceae_uncultured | 6.01 | 5.96 | 3.64 | 1.71 | 2.96 | 3.67 |  |
| Bacillales |  |  | 1.44 | 1.24 | 0.72 | 5.67** | unclassified_Prevotellaceae | 4.28 | 4.89 | 4.16 | 3.71 | 3.44 | 3.87 |  |
| Escherichia-Shigella-Hafnia |  |  |  |  | 18.35 | 0.46** | no_rank_Ruminococcaceae_uncultured | 4.06 | 3.5 | 4.58 | 4.71 | 6.33 | 5.86 |  |
| Helicobacter | 1.39 | 1.34 |  |  | 0.9 | 0.9 | no_rank_S24-7 | 3.9 | 3.59 | 4.79 | 4.86 | 6.52 | 5.82 |  |
| Flavobacterium | 10.78 | 1.24** | 0.48 | 7.41** |  |  | Treponema | 3.72 | 4.14 | 2.59 | 5.02 | 3.85 | 9.54 |  |
| Chryseobacterium | 2.01 | 0.88* |  | 1.27 |  |  | Bacteroidales | 2.74 | 3.26 | 2.8 | 3.02 | 3.37 | 3.42 |  |
| Lachnospiraceae |  |  | 1.2 | 0.58 |  |  | Rikenellaceae | 2.64 | 2.33 | 3.06 | 2.68 | 3.47 | 2.91 |  |
|  |  |  |  |  |  |  | Parabacteroides | 1.46 | 1.58 |  | 1.5 |  | 1.69 |  |

NT: non-transgenic pigs, T: transgenic pigs,*P<0.05; **P<0.01, by Mann-Whitney U test.
