# Supplementary material for: Safety Evaluation of Neo Transgenic Pigs by Studying Changes in Gut Microbiota Using High-Throughput Sequencing Technology
Source: PLoS One. 2016 Mar 11;11(3):e0150937. doi: 10.1371/journal.pone.0150937 (PMC4788350; doi:10.1371/journal.pone.0150937)
Supplement: S4 Table — (DOCX) [file pone.0150937.s012.docx]

**S4 Table. Overview of pyrosequencing results of Group A fecal samples collected at various time points post antibiotic feeding**

| Sample ID | Reads | OUT (97%) | ace | chao | coverage | Sample ID | Reads | OUT (97%) | ace | chao | coverage |
| --- | --- | --- | --- | --- | --- | --- | --- | --- | --- | --- | --- |
| 0D-1 | 7822 | 2697 | 25803 | 11312 | 0.98 | 23D-1 | 7144 | 3174 | 23282 | 12318 | 0.98 |
| 0D-2 | 12047 | 5542 | 48402 | 22349 | 0.98 | 23D-2 | 9308 | 5247 | 60151 | 25807 | 0.97 |
| 0D-3 | 13853 | 4490 | 33497 | 16180 | 0.99 | 23D-3 | 7781 | 3978 | 39872 | 17849 | 0.98 |
| 0D-4 | 10986 | 3104 | 23324 | 11807 | 0.98 | 23D-4 | 9908 | 5235 | 57703 | 23699 | 0.96 |
| 0D-5 | 11206 | 4676 | 44201 | 18753 | 0.99 | 23D-5 | 8892 | 4721 | 40328 | 19837 | 0.98 |
| 0D-6 | 10593 | 4807 | 41668 | 18627 | 0.98 | 23D-6 | 9686 | 3672 | 32423 | 14744 | 0.98 |
| 0D-7 | 7598 | 3603 | 39364 | 17008 | 0.98 | 23D-7 | 6673 | 3935 | 47225 | 20132 | 0.96 |
| 0D-8 | 7154 | 3560 | 36651 | 15293 | 0.98 | 23D-8 | 8221 | 4546 | 54074 | 24356 | 0.97 |
| 8D-1 | 11349 | 4819 | 45232 | 20686 | 0.98 | 45D-1 | 9899 | 4978 | 49104 | 21556 | 0.98 |
| 8D-2 | 9541 | 4425 | 42822 | 20046 | 0.98 | 45D-2 | 9456 | 4810 | 55098 | 22103 | 0.98 |
| 8D-3 | 11044 | 4951 | 48116 | 21903 | 0.98 | 45D-3 | 11196 | 5292 | 57741 | 24683 | 0.96 |
| 8D-4 | 10435 | 4394 | 40367 | 19069 | 0.97 | 45D-4 | 7493 | 3953 | 41974 | 19305 | 0.97 |
| 8D-5 | 10274 | 4629 | 42532 | 19665 | 0.98 | 45D-5 | 10397 | 4949 | 49506 | 21261 | 0.98 |
| 8D-6 | 10308 | 5096 | 44085 | 19162 | 0.98 | 45D-6 | 10749 | 5350 | 51150 | 23428 | 0.98 |
| 8D-7 | 6748 | 3373 | 38424 | 16815 | 0.98 | 45D-7 | 7061 | 3985 | 47880 | 20832 | 0.96 |
| 8D-8 | 8656 | 4171 | 42409 | 19359 | 0.98 | 45D-8 | 7457 | 3855 | 45212 | 19830 | 0.97 |

-1, -2, -3, and -4 refer to non-transgenic pigs; -5, -6, -7 and -8 refer to transgenic pigs.
