# Supplementary material for: Safety Evaluation of Neo Transgenic Pigs by Studying Changes in Gut Microbiota Using High-Throughput Sequencing Technology
Source: PLoS One. 2016 Mar 11;11(3):e0150937. doi: 10.1371/journal.pone.0150937 (PMC4788350; doi:10.1371/journal.pone.0150937)
Supplement: S5 Table — (DOCX) [file pone.0150937.s013.docx]

**S5 Table. Overview of pyrosequencing results of Group B fecal samples collected from different intestinal sections**

| Sample ID | Reads | OUT (97%) | ace | chao | coverage | Name | Reads | OUT (97%) | ace | chao | coverage |
| --- | --- | --- | --- | --- | --- | --- | --- | --- | --- | --- | --- |
| Duo-1 | 9730 | 2004 | 14288 | 7688 | 0.99 | Cec-1 | 8708 | 3404 | 35958 | 16236 | 0.98 |
| Duo-2 | 11854 | 2627 | 19969 | 10139 | 0.98 | Cec-2 | 10146 | 3957 | 35695 | 16497 | 0.98 |
| Duo-3 | 10607 | 2639 | 28625 | 12077 | 0.98 | Cec-3 | 8071 | 3592 | 32799 | 15898 | 0.98 |
| Duo-4 | 10372 | 2428 | 19614 | 9554 | 0.99 | Cec-4 | 8636 | 2864 | 28428 | 12766 | 0.98 |
| Duo-5 | 11009 | 3013 | 22759 | 11131 | 0.98 | Cec-5 | 8183 | 3698 | 36080 | 16584 | 0.98 |
| Duo-6 | 9372 | 2859 | 20551 | 10491 | 0.98 | Cec-6 | 8002 | 3374 | 38254 | 17736 | 0.98 |
| Duo-7 | 11840 | 2546 | 22115 | 10124 | 0.98 | Cec-7 | 6940 | 3235 | 37072 | 16176 | 0.97 |
| Duo-8 | 13532 | 4640 | 36025 | 18030 | 0.98 | Cec-8 | 8566 | 3605 | 33986 | 14937 | 0.97 |
| Duo-9 | 11233 | 2588 | 18137 | 9018 | 0.99 | Cec-9 | 8134 | 4321 | 44434 | 20035 | 0.97 |
| Duo-10 | 8742 | 1805 | 15959 | 7297 | 0.98 | Cec-10 | 9166 | 3961 | 35544 | 17372 | 0.98 |
| Jej-1 | 9239 | 2236 | 19976 | 8453 | 0.98 | Col-1 | 7373 | 3481 | 38518 | 16758 | 0.97 |
| Jej-2 | 10879 | 2999 | 27676 | 13319 | 0.98 | Col-2 | 8392 | 3850 | 43392 | 19211 | 0.97 |
| Jej-3 | 11403 | 2411 | 21889 | 10023 | 0.98 | Col-3 | 7890 | 3025 | 30564 | 12915 | 0.98 |
| Jej-4 | 12736 | 1488 | 16130 | 6695 | 0.99 | Col-4 | 7989 | 3123 | 33207 | 14020 | 0.97 |
| Jej-5 | 14197 | 2720 | 19850 | 10549 | 0.99 | Col-5 | 8429 | 3716 | 39192 | 16594 | 0.98 |
| Jej-6 | 11002 | 2365 | 21385 | 9626 | 0.99 | Col-6 | 8038 | 3488 | 37241 | 15739 | 0.98 |
| Jej-7 | 9827 | 2614 | 22903 | 10564 | 0.98 | Col-7 | 7457 | 3083 | 35413 | 15046 | 0.97 |
| Jej-8 | 12189 | 3788 | 29285 | 14616 | 0.98 | Col-8 | 9091 | 3747 | 36608 | 15362 | 0.98 |
| Jej-9 | 13527 | 4011 | 31057 | 14274 | 0.99 | Col-9 | 7757 | 3987 | 39408 | 16737 | 0.97 |
| Jej-10 | 12614 | 2056 | 15580 | 7612 | 0.99 | Col-10 | 10403 | 4700 | 43546 | 20129 | 0.98 |
| Ile-1 | 8863 | 2484 | 17482 | 8469 | 0.98 | Rec-1 | 7744 | 3557 | 40630 | 16126 | 0.97 |
| Ile-2 | 8745 | 2490 | 22594 | 11190 | 0.98 | Rec-2 | 8743 | 3538 | 34539 | 16243 | 0.98 |
| Ile-3 | 11601 | 2009 | 16438 | 8273 | 0.99 | Rec-3 | 9519 | 3163 | 35220 | 14160 | 0.98 |
| Ile-4 | 11209 | 2765 | 20395 | 10300 | 0.99 | Rec-4 | 9302 | 4221 | 38623 | 16615 | 0.98 |
| Ile-5 | 12120 | 3181 | 25027 | 11340 | 0.99 | Rec-5 | 8232 | 3787 | 40948 | 19417 | 0.98 |
| Ile-6 | 11480 | 2171 | 19510 | 9230 | 0.99 | Rec-6 | 8376 | 3745 | 45424 | 18803 | 0.98 |
| Ile-7 | 9412 | 2322 | 19816 | 10080 | 0.98 | Rec-7 | 6543 | 3024 | 31982 | 14031 | 0.97 |
| Ile-8 | 13738 | 3834 | 27181 | 13437 | 0.98 | Rec-8 | 8620 | 3626 | 41526 | 17035 | 0.97 |
| Ile-9 | 11190 | 2726 | 19106 | 9192 | 0.99 | Rec-9 | 9564 | 4380 | 44324 | 19436 | 0.97 |
| Ile-10 | 13048 | 1975 | 12796 | 6581 | 0.99 | Rec-10 | 6563 | 3137 | 34167 | 14369 | 0.97 |

-1, -2, -3, -4, and -5 refer to non-transgenic pigs; -6, -7, -8, -9 and -10 refer to transgenic pigs.
